# 

**Published:** 1898-05

**Authors:** 


### CONTENTS.

PROCEEDINGS.

St. Augustine Meeting—Abstracts ........ 201

COMMUNICATIONS.

Personal Experiences vs. "Scientific Proof," as Factors Bearing Upon the Amalgam Question ........ 213

SELECTIONS.

A Wineglass of Vinegar ........ 212
Oil of Eucalyptus to Cure Chilblains ........ 237
Japan ........ 237
Dr. H. T. Whitney ........ 242

NOTICES.

Second Tri-State Dental Meeting, Hotel Victory, Put-in-Bay, Lake Erie, Ohio, June 21st, 22d, 23d, 1898 ........ 237
Program of the Section on Stomatology of the American Medical Association at Denver, Col., June 7-10, 1898 ........ 240

EDITORIAL

American Medical Association—Dental Section ........ 240
Transfer of Students ........ 242
The First Subscribers to the First Dental Journal ........ 243

